# Potential Application of Tregitopes as Immunomodulating Agents in Multiple Sclerosis

**DOI:** 10.1155/2011/256460

**Published:** 2011-09-15

**Authors:** Wassim Elyaman, Samia J. Khoury, David W. Scott, Anne S. De Groot

**Affiliations:** ^1^Center for Neurologic Diseases, Brigham and Women's Hospital and Harvard Medical School, 77 Avenue Louis Pasteur, NRB 641, Boston, MA 02115, USA; ^2^Uniformed Services University of the Health Sciences, Bethesda, MD 20814, USA; ^3^EpiVax Inc., University of Rhode Island, Providence, RI 02903, USA

## Abstract

The induction of immunologic tolerance is an important clinical goal in autoimmunity. CD4^+^ regulatory T (Treg) cells, defined by the expression of the transcription factor forkhead box P3 (FoxP3), play a central role in the control of autoimmune responses. Quantitative and qualitative defects of Tregs have been postulated to contribute to failed immune regulation in multiple sclerosis (MS) and other autoimmune diseases. This paper highlights the potential uses of T regulatory cell epitopes (Tregitopes), natural Treg epitopes found to be contained in human immunoglobulins, as immunomodulating agents in MS. Tregitopes expand Treg cells and induce “adaptive Tregs” resulting in immunosuppression and, therefore, are being considered as a potential therapy for autoimmune diseases. We will compare Tregitopes versus intravenous immunoglobulin (IVIg) in the treatment of EAE with emphasis on the potential applications of Tregitope for the treatment of MS.

## 1. Multiple Sclerosis and the Adaptive Immunity

Multiple sclerosis (MS) affects over 2 million people worldwide and is the leading cause of neurological disability in young adults. It is now clear that the core process in MS is inflammatory, with myelin-reactive T helper (T_H_) cells and their mediators triggering injury of axons and their myelin sheaths through a complex sequence of events [1]. Experimental autoimmune encephalomyelitis (EAE) has been used as a model for MS for more than 40 years and has been a major factor in determining the path of MS research. In EAE, T_H_1 and T_H_17 effector cells, major producers of IFN*γ* and IL-17, respectively, have been associated with the disease cascade that causes encephalitogenicity [2–6]. The observation that IFN*γ* and IL-17 expression were upregulated in peripheral circulating T cells as well as in the central nervous system of MS patients gave validity to the hypothesis that T_H_1 and T_H_17 cells were potentially pathogenic in MS patients [7–11]. Thus, current therapies for MS are immunomodulatory and have been effective in decreasing relapse rates but seemingly far less effective in preventing disease progression, defined as an accumulation of neurologic disability. 

Although immune dysregulation had been described in MS patients for some time, a major breakthrough came in the 1990s with the discovery of a specific subtype of CD4^+^CD25^+^ suppressor T cells (now called regulatory T cells or Tregs) [12]. Treg cells are a specialized subpopulation of T cells that act to suppress activation of undesirable immune responses and thereby maintain immune system homeostasis and tolerance to self-antigens. At least two major subtypes of Tregs have been identified: natural Tregs (nTregs) generated in the thymus and inducible Tregs (iTregs) generated in the periphery from CD4^+^CD25^−^FoxP3^−^ effector T cells. Almost a decade after their discovery, the Hafler group described first a functional defect of peripheral CD4^+^CD25^+^ Tregs in patients with relapsing-remitting MS [13] that was followed by several reports confirming these observations in MS patients [14, 15]. Thus, therapy that restores impaired nTreg cell homeostasis while suppressing pathogenic effector T cells (T_H_1 and T_H_17) at the right time and more importantly at the right place will be a promising approach in MS patients. Adoptive cell transfer of patient-specific CD4^+^CD25^+^ Tregs has been considered a potential therapeutic approach [16]. Strategies aimed at expanding Tregs in patients with autoimmune diseases are viewed as promising. The technical barrier in translating this strategy to clinical practice is to find safe and effective method to induce Tregs and suppress or convert effector cells to adaptive Tregs in the target organs in autoimmune diseases.

## 2. Discovery of Tregitopes

**Figure 1:**
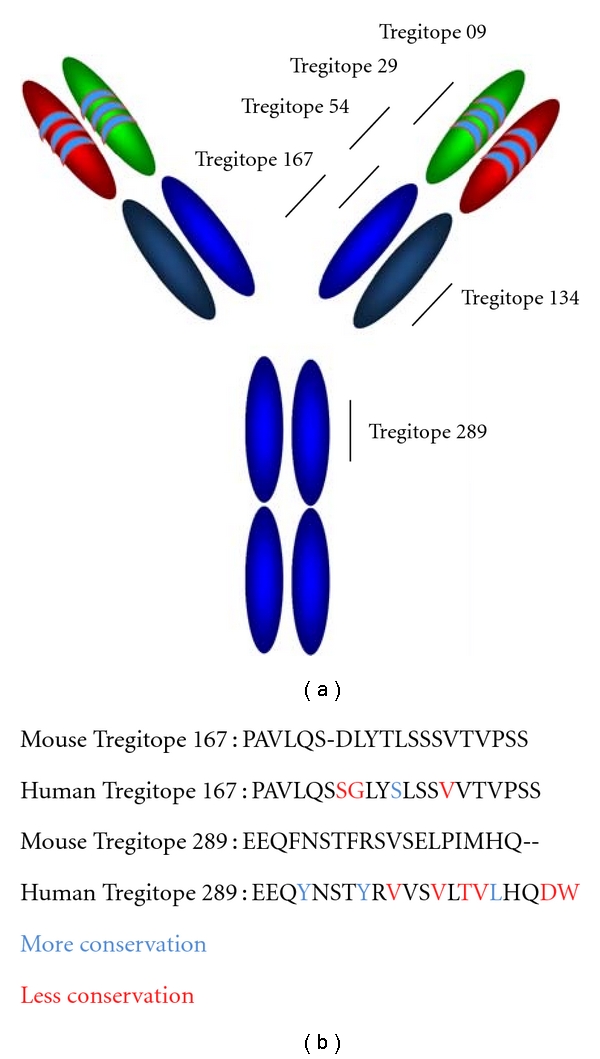
(a) Approximate location of IgG “Tregitopes” EpiVax murine and human Tregitope peptides. (b) Human and mouse Tregitopes are highly conserved. The sequences in bold are considered the core Tregitope sequence and differences are color coded.

T regulatory cell epitopes (Tregitopes) were discovered when the team of De Groot et al. [17] was searching for potential effector T-cell epitopes in monoclonal antibodies and uncovered several strong signals for T cell responses in the Fc and Fab domains of IgG antibodies. To identify these epitopes, they used EpiMatrix, an epitope mapping tool, and ClustiMer, a promiscuous epitope mapping tool [18]. These putative T-cell epitope sequences were highly conserved across IgG isotypes and in published IgG sequence databases, suggesting that they were functional (Figure 1). Indeed, the peptides representing these highly conserved, promiscuous regions appeared to suppress immune responses in coculture and the expanded cells exhibited surface marker characteristics and the cytokine profile of Tregs [17]. Tregitopes are peptides that have the following four characteristics: (i) their sequences are highly conserved in similar autologous proteins, (ii) they almost all exhibit “EpiBars” or a pattern (as measured by EpiMatrix) that suggests promiscuous MHC binding [19], (iii) T cells responding to these Tregitopes exhibit a T regulatory phenotype (CD4^+^CD25**^+^**FoxP3^+^) and secrete IL-10, TGF-*β* and MCP-1 ([17] and unpublished observations), and (iv) coincubation of Tregitopes with immunogenic peptides inhibits T cell proliferation in vitro and suppresses the secretion of effector cytokines and chemokines in response to the immunogenic peptides. 

Prior to the discovery of Tregitopes, no Treg cells that respond to Ig epitopes had been identified nor had nTregs reacting to Ig been used to induce adaptive tolerance. We have proposed that Tregitope recognition by Tregs initiates a series of events that culminate in (i) suppression of effector T cell immune responses in the immediate vicinity of the activated Treg (bystander suppression) and/or (ii) induction of antigen-specific iTregs which downregulate immune responses to a given antigen. 

A description of the initial two Tregitopes (289 and 167, both in the heavy chain of IgG) was published in Blood in 2008 [17]. When added to a culture of freshly isolated human peripheral blood mononuclear cells ex vivo, these Tregitopes led to an expansion of the number of Tregs and/or an upregulation of FoxP3 expression in previously FoxP3-negative T cells. We also demonstrated (i) induction of natural Tregs in a four day incubation and (ii) a phenotype change in effector T cells incubated with the Tregitopes away from IL-5 secreting cells to null cells and increased expression of adaptive Treg cell surface proteins (GITR and CTLA-4) [17]. Coadministration of the Tregitopes in vivo with dust mite antigens suppresses immune response to the antigens, and this response is partially dependent on the presence of regulatory T cells (as defined by the cell surface markers CD4^+^ and CD25^hi^ and intracellular FoxP3). 

### 2.1. Tregitopes and Tolerance

It has become increasingly clear that CD4^+^CD25^+^ regulatory T cells are an important component of immune regulation in the periphery. Autoreactive T cells with moderate affinity may escape thymic deletion and be converted to function as effector cells or “natural” regulatory T cells. These moderate binding Treg cells are exported to the periphery, where they provide a source of protective immunity against foreign antigens or suppression of immunity against self-antigens. It has been suggested that T cells must be tolerant to Ig molecules that have undergone somatic hypermutation following primary engagement of the variable region with an antigen [20]. Indeed, it has been observed that tolerance induction in a murine diabetes model using delivery of Fc fusion proteins in B cells is due to induction of regulatory T cells [21].

### 2.2. Dendritic Cells (DCs)

Dendritic cells are essential to generate and maintain immunological tolerance. They are critical intermediaries between antigens and lymphocytes. DCs sample peripheral antigens in the skin, gastrointestinal and respiratory epithelia, migrate to the T cell areas of lymphoid tissue, where they activate and expand antigen-specific helper and killer T cells [22]. They are known as “professional antigen presentation cells”, because they efficiently process and present antigen-derived peptides in the context of MHC. Respectively, CD8^+^ and CD4^+^ T cells recognize MHC I: peptide and MHC II: peptide complexes and initiate the adaptive immune response [23, 24]. In addition to their role as mediators of immune responses, DCs play a critical role in the induction of regulatory T cells [25]. DC-SIGN (dendritic cell-specific intercellular adhesion molecule-3-grabbing nonintegrin), a C-type lectin mainly present at the surface of immature dendritic cells, plays a relevant role activating and tailoring adaptive immune responses against different pathogens. This lectin recognizes, in a multivalent and calcium-dependent manner, highly glycosylated proteins present at the surface of pathogens [26]. In studies carried out by Anthony et al. in the Ravetch group [27], tolerance was induced following treatment of collagen-induced arthritis with sialylated intravenous immunoglobulin (IVIg) Fc fragments. The efficacy of sialylated-Fc, which is believed to be more efficiently taken up by DC-SIGN could be explained by the presence of Tregitope in the Fc. However, in the Ravetch studies, tolerance induction required Fc-sialylation, which has not been required for studies carried out by Khoury and Elyaman and De Groot et al. [28] and [17]). Tregitopes administered in saline are able to suppress immune response to antigen, and the affinity of binding to HLA correlates with their suppression ability suggesting that the natural receptor for Tregitopes contained in IgG Fc is the human HLA molecule. 

One explanation that may tie the two observations together is that DC-SIGN may enable the trafficking of sialylated IgG to the antigen processing and presentation pathway. The requirement for sialylation does not explain the induction of natural and inducible Tregs following administration of Tregitope peptides in saline [17]. Our findings are strengthened by reports that polyclonal immunoglobulin therapies induce expansion of Tregs and IL-10 secretion in vivo in animals and humans [29–31]. Others have described the immunosuppressive effects of non-Fc IgG-derived peptides (included in our list of Tregitopes [23, 32, 33] providing independent confirmation of the hypothesis.

## 3. Tregitopes versus IVIg

The important discovery of Tregitopes has the potential to bring understanding about a number of phenomena related to Ig, including tolerance to antibody (Ab) variable regions, the tolerogenic properties of immunoglobulin-antigen (Ag) conjugates, the weak immunogenicity of Fc fusion proteins, and the therapeutic and regulatory effects of clinical preparations of intravenous immunoglobulin (IVIg) on autoimmune and inflammatory diseases. Immunoglobulin (Ig) has long been known to have tolerogenic properties. Thus, Ags conjugated to Ig elicit tolerance rather than immunity, and intravenous administration of pooled Ig from multiple donors, known as IVIg, is used in clinical practice to treat autoimmune and inflammatory diseases.

**Figure 2:**
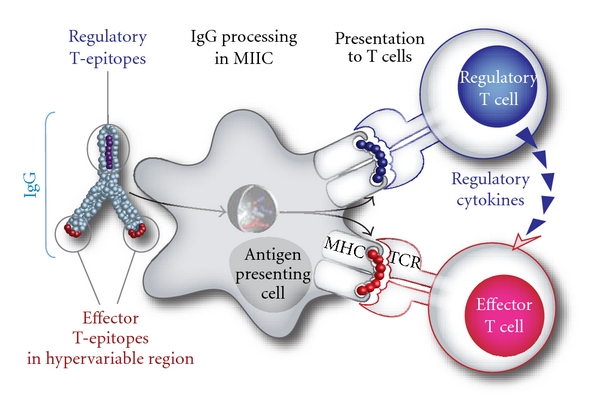
Proposed role of Tregitopes in IgG. Adapted with permission from de Groot et al. [18]. Antibody-derived Treg epitope (dark blue) activated regulatory T cells (Treg), which leads to suppression of effector T cells (Teff) that recognize effector epitope (red), like those of IgG hypervariable regions to which central tolerance does not exist.

The presence of Tregitopes in IgG may explain the induction of tolerance with intravenous IgG (Figure 2). Even though the role of immunoglobulin in tolerance was postulated in studies published almost a century ago [34], the mechanism behind antibody-mediated immune suppression has remained unclear. Some studies have shown that the Fab region is as capable of inducing suppression as well as intact antibodies with an Fc region (which would be consistent with our discovery of Tregitopes in both the Fab and Fc regions) [35], while other studies indicate that Fc, Fab, and intact IgG was incapable of immune suppression [36]. In some cases, the authors postulated that the immune suppression may be due to the interaction of the Fc domain with yet to be discovered Fc*γ* receptors, and others have concluded that the effect is due to Fc-independent mechanisms such as epitope masking [36], while still others provide only a broad explanation wherein the type of immune response (effector or tolerance) to a given antibody idiotype is attributed to the isotype of the antibody and the potential immunogenicity of their idiotypes [37]. Of note, Kessel et al. recently showed that IVIg “improved the suppressive function” of nTregs [38].

The presence or absence of Tregitopes has been associated with immune responses to monoclonal antibodies in clinical studies. Immunogenicity occurs despite “humanization” of antibodies as demonstrated in [18]. Indeed, a careful review of monoclonal antibody immunogenicity in clinical practice has revealed a correlation between the presence of hTregitopes and lower immunogenicity of monoclonal antibodies in human studies; a significant (*P* < 0.002) correlation was found between Tregitope content and lower reported immunogenicity (reported in De Groot and Martin's analysis of 21 monoclonals in current clinical use [18]).

This model does not ignore the contribution of Fc receptors to IgG-mediated anti-inflammatory processes. Fc-gamma Receptors (Fc*γ*R) are required for rapid uptake of IgG and immune complexes into antigen-presenting cells during the initial inflammatory phase, and the inhibitory Fc receptor, Fc*γ*RIIb, increases the threshold for cell activation during the refractory phase of immune response. In our model, Tregitope activation of Treg would stimulate the release of cytokines such as IL-4 and IL-10 that are known to shift expression from the activating Fc*γ*RI and Fc*γ*RIIa to Fc*γ*RIIb [33].

Immunization with antigens fused to the IgG Fc region is now a well-established method of tolerizing against the antigen. For example, Baxevanis et al. evaluated the effect of administering human Fc (hFc) to mice in 1986 [39], causing tolerance rather than antihuman immune response. These studies were eventually replicated by Scott et al., who showed that (i) fusion of an IgG heavy chain to antigen, or administration of the Fc region in conjunction with the antigen, could induce tolerance, (ii) MHC class II molecules were required for induction of tolerance [40], and (iii) the human Fc region plays an essential role in immune suppression by IgG fusion, but Fc binding is not required [41]. Furthermore, mice have homologous T regulatory epitopes, which may explain earlier observations that Fc [39] and Fc-protein fusions [42] stimulate a tolerizing immune response.

## 4. Tregitopes in Autoimmunity

The discovery of Tregitopes inspired a reconsideration of published research on IVIg and Fc-fusions. The Tregitope hypothesis may change the interpretation of work published almost a century ago [34] as well as more recent studies associating an immunosuppressive effect with the Fc fragment of IgG. Preliminary studies generated by Khoury and colleagues provided proof that stimulation of antigen-specific T cells in the presence of the IgG-derived Tregitope-specific natural Tregs induces adaptive tolerance to the myelin antigen-mediated autoimmune encephalomyelitis by tipping the immune response toward anti-inflammatory phenotype [28].

A study published by Ephrem et al. in Blood also demonstrated that IVIg therapy induced expansion of Tregs and protected against development of EAE induced by active immunization with myelin oligodendrocyte glycoprotein (MOG)35–55 [43]. IVIg has been considered as a potential systemic therapy for MS and other autoimmune diseases [44, 45]; however, the use of human IVIg is associated with a number of real and potential adverse effects [46, 47]. In order to explore a safer, more effective alternative to IVIg for the treatment of MS, we have evaluated the capacity of IgG-derived Tregitopes to generate antigen-specific adaptive tolerance induction to MOG35–55 epitopes in vivo. Our findings point to a tolerogenic effect of Tregitopes coadministration on immune responses to the MOG35–55 epitopes in vitro and in vivo, a result consistent with the results of Zaghouani and coworked using IgG fusion proteins with CNS antigens [48]. The success of Tregitopes in suppressing experimental autoimmunity may lead to their use as a therapy for MS. Successful development of Tregitope therapy would have a radical impact on the fields of autoimmunity, transplantation, and protein therapeutics and may lead to development of an alternative to IVIg.

## 5. Conclusion

Is there a role for Tregitope in future clinical practice? Emerging approaches to autoimmune disease treatment currently involve induction of Tregs using monoclonal antibodies (mAbs) such as anti-CD3 (Teplizumab, Macrogenics, Otelixizumab, and Tolerx), which induce Treg cells. Anti-CD3 treatment has shown some efficacy in human studies, but the mechanism of Treg induction is elusive and the effect appears to be short lasting [49, 50]. Antigen specificity and localized immunosuppressive effects are believed to be advantages of the Tregitope approach that might reduce side effects (such as infections) associated with more broadly suppressive treatments. Our prior studies indicate that the effect of Tregitopes may be long lasting in mice (100 days in the transplant model, and up to 30 weeks in NOD mice (De Groot et al., submitted), but maintenance of tolerance in humans may require “booster” treatments and/or intermittent low-dose IL-2 to maintain Treg populations [51]. Induction of tolerance using Tregitope therapy would alleviate the burden of repeated and long-term medical interventions associated with chronic autoimmune diseases. The use of Tregitopes may be a safe and effective approach to expand natural Tregs in autoimmune diseases such as MS and in transplantation.
